# Craniofacial and Dental Abnormalities in Children With Intrauterine Growth Restriction: A Comparative Cross-Sectional Study

**DOI:** 10.7759/cureus.96955

**Published:** 2025-11-16

**Authors:** Haritha Nimmagadda, Manisha Mishra, Shobha Verma, Sunita P Bharti

**Affiliations:** 1 Anatomy, Mahatma Gandhi Mission (MGM) Medical College, Nerul, Navi Mumbai, IND

**Keywords:** cephalometric analysis, craniofacial morphometry, enamel defects, intrauterine growth restriction (iugr), pediatric dentistry, small for gestational age (sga)

## Abstract

Background: Intrauterine growth restriction (IUGR) has been linked to disturbances in growth after birth and various developmental anomalies. However, its impact on the shape of the face and dental health is still a question. Exploring these links can be helpful for the early prevention and orthodontic treatment of a pediatric population.

Objective: Comparison of differences in craniofacial morphometric parameters and dental abnormalities of children with IUGR and healthy children.

Methods: A cross-sectional case-control study was performed in 60 children aged between 3 and 7 years (30 IUGR; 30 controls matched for age and sex). Lateral cephalometric radiograph standardization was done for assessment of craniofacial parameters, and dental anomalies were confirmed by clinical examinations. The statistical analysis consisted of Student’s t-test, chi-square test, and logistic regression with significance set at p <0.05.

Results: Children with IUGR had a markedly reduced cranial base length (91.3 ± 4.9 mm vs. 99.6 ± 5.4 mm, p < 0.001) and mandibular length (65.2 ± 4.0 mm vs. 73.9 ± 4.5 mm, p < 0.001) and an increased lower facial height (54.6 ± 3.1 mm vs. 51.0 ± 3.2 mm, p < 0.001). Enamel defects in the IUGR group (33.3%) were twice as many as in the control group (10.0%), and the adjusted odds were almost five times higher (OR = 4.83, 95% CI 1.04 - 22.36, p = 0.044). The occurrences of caries, hypodontia, and microdontia in the IUGR group have increased, but their differences are not statistically significant.

Conclusion: IUGR is related to changed patterns of craniofacial development that feature shortening of the cranial base and mandibular lengths and lengthening of the lower facial height. It is also associated with a greater number of cases of enamel defects. Screening of children with IUGR and their cephalometric evaluation at an early stage will provide better preventive and orthodontic interceptive treatment guidance.

## Introduction

Intrauterine growth restriction (IUGR) refers to the condition whereby a fetus does not reach the growth potential set by its genes and is usually determined as a birth weight or length of less than the 10th percentile for gestational age [1]. IUGR accounts for a large proportion of perinatal morbidity and mortality and may also affect somatic, cognitive, and metabolic development over the course of childhood [2,3]. On average, the worldwide prevalence of IUGR is between 5% and 10% of live births; however, the rate is higher in developing countries due to factors such as maternal malnutrition, placental insufficiency, and socio-economic inequalities [2,3].

The development of the craniofacial region involves a series of stages controlled by genes, nutrition, and various environmental factors, which all occur during intrauterine life. Any disruption of these pathways, like the one caused by IUGR, could result in changed skeletal proportions and altered dentoalveolar relationships, and thus, children suffering from this condition may become imbalanced in maxillofacial and occlusal harmony [4,5]. Various cephalometric and clinical investigations have revealed that children with growth restriction exhibit the tendency of having a shorter cranial base and mandibular length, increased lower facial height, and facial proportions different than those of their age-matched counterparts [6].

Aside from skeletal modifications, dental anomalies have also been linked with IUGR. Oftentimes, enamel hypoplasia, microdontia, and delayed eruption are among the symptoms of children who have been born with low birth weight or growth restriction [7,8]. Pinho et al. found that a significant relationship exists between low birth weight and enamel developmental defects [7], whereas Garmash and Ryabokon observed the incidence of dental health problems to be higher in the pediatric population with a history of symmetrical IUGR [8]. The results emphasize the importance of prenatal growth conditions for oral and craniofacial health.

While recognizing these changes, there are still a few quantitative studies that simultaneously analyze craniofacial morphometry and dental abnormalities in IUGR populations, especially in India. A thorough comprehension of these morphological changes can not only facilitate orthodontic preventive and interceptive measures but also help pediatric dental practitioners in early diagnosis and management.

The current research has been primarily focused on assessing the craniofacial morphometric changes of the head and face between the IUGR children and the healthy ones. As secondary outcomes, dental abnormalities such as enamel defects, hypodontia, and microdontia were intended to be recorded prevalently. Besides that, the investigation aimed to offer the data of an Indian pediatric cohort, where the evidence is very limited, and to unfold the clinical implications for early orthodontic screening and growth monitoring in children with IUGR.

## Materials and methods

This cross-sectional case-control investigation took place at the Department of Preventive and Pediatric Dentistry in cooperation with the Departments of Anatomy and Histology from January 2023 to December 2023. The main objective was to compare craniofacial morphometric parameters and dental anomalies in a group of children with a confirmed history of intrauterine growth restriction (IUGR) versus healthy age-matched children. Approval for the study was granted by the Institutional Ethics Committee (Approval No. MGM/IEC/2023/47; dated 12 January 2023). Consent from the parents or legal guardians was obtained in writing prior to participation, and the investigation was carried out in accordance with the ethical principles set out in the Declaration of Helsinki (2013 revision) [8].

Thirty children with confirmed IUGR and 30 healthy children matched for age and sex, sixty children aged three to seven years in total, were the subjects of this study. The participants were selected using consecutive sampling from the outpatient pediatric dentistry clinic to reduce selection bias [9]. The criteria for inclusion in the test group were children whose perinatal records showed birth weight less than the 10th percentile for gestational age or who had a recorded diagnosis of IUGR. The control group consisted of full-term children with normal birth weights and no systemic or developmental abnormalities. Those with nutritional deficiencies, known systemic diseases that affect growth, congenital craniofacial syndromes, neurological or chromosomal disorders, or those whose parents did not consent were excluded. Besides this, demographic and socioeconomic data were gathered for all the children, such as gestational age, birth weight, order of birth, and family background [9].

One-to-one matching was done for each IUGR participant with a control child of the same sex and similar chronological age (±6 months), both of them being consecutively recruited from the same pediatric dental outpatient department. The comparability of the socioeconomic background was also checked. The average age between the two groups was not significantly different (p = 0.94), and the sex distribution was the same (p = 1.00). While small personal growth differences cannot be excluded, this method minimized the bias of the demographic.

Two calibrated pediatric dental specialists performed a thorough dental extraoral and intraoral examination for each subject. The study also included an examination of the developmental dental anomalies such as hypodontia, microdontia, supernumerary teeth, enamel hypoplasia, fluorosis, caries, and other structural defects according to the World Health Organization’s Oral Health Surveys: Basic Methods (5th edition) [10]. The examiners were trained on 10 preliminaries that were not part of the final sample, and inter-examiner agreement was measured by Cohen's kappa statistic, which indicated κ = 0.86, representing an excellent level of agreement [11].

All subjects had standard lateral cephalometric radiographs taken with the Sirona ORTHOPHOS XG Plus system. The pictures were taken with the patient sitting upright in a natural head position, the teeth in centric occlusion, and the lips relaxed, according to the imaging principles given by Jacobson and Jacobson [12]. The cephalometric landmarks Sella, Nasion, Point A, Point B, Gonion, Menton, Gnathion, and Pogonion were manually traced by one trained examiner to measure the length of the cranial base, mandible, and lower facial height. To check the consistency of the findings, every single measurement was done a second time after two weeks, and the intra-observer agreement was determined by the intra-class correlation coefficient (ICC = 0.93).

Statistical analysis of all data was done through IBM Corp. Released 2017. IBM SPSS Statistics for Windows, Version 26.0. Armonk, NY: IBM Corp. Continuous variables were described by means of mean ± standard deviation, and categorical data were presented as frequencies and percentages. Based on an a priori power analysis (α = 0.05, two-tailed), 26 participants per group were needed to identify large morphometric effects (Cohen’s d = 0.80) with 80% power. Thirty per group (N = 60) were therefore recruited, resulting in a power of ~86%. The Student’s t-test was used to compare continuous craniofacial variables between the two groups, while the chi-square or Fisher’s exact test, depending on the data, was used to determine the relationship between categorical variables such as the presence of enamel defects or hypodontia. To determine the independent factors that can predict the occurrence of enamel defects, binary logistic regression analysis was conducted, yielding odds ratios (OR) together with 95% confidence intervals (CI). The significance level of the test was set at p < 0.05. The data met the requirements for parametric analyses, as normality (Shapiro-Wilk test) and homogeneity of variance (Levene’s test) were checked and confirmed. Effect sizes were determined by Cohen’s d for continuous variables and by Cramér’s V for categorical variables [13].

## Results

**Figure 1:**
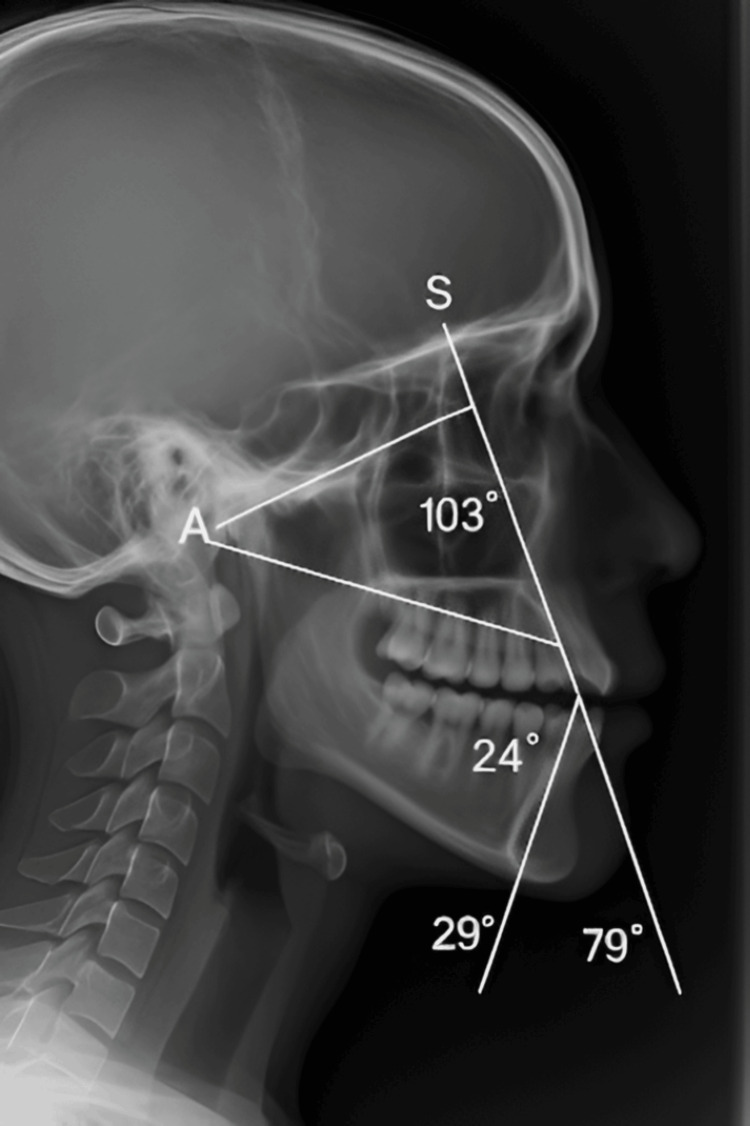
Anteroposterior cephalogram demonstrating transverse craniofacial relations and maxillomandibular alignment. Key cephalometric landmarks (S, N, ANS, PNS, Go, Me, Gn, and Pog) are indicated. Lateral cephalometric radiograph with cephalometric landmarks marked for sagittal and vertical analysis. S: Sella; N: Nasion; A: Point A (subspinale); B: Point B (supramentale)

**Figure 2:**
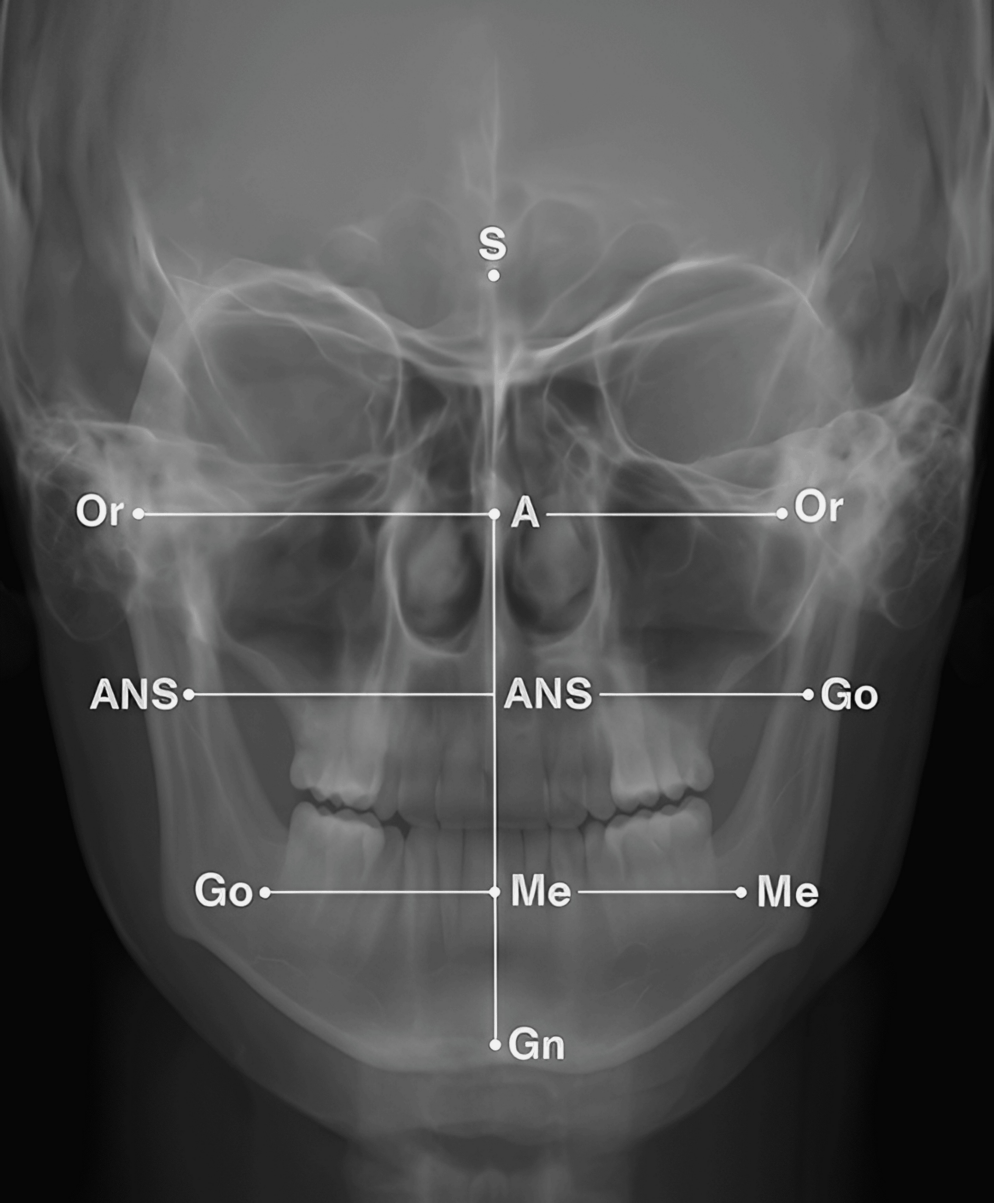
Frontal cephalometric radiograph showing key anatomical landmarks used in morphometric analysis. S: Sella; N: Nasion; Or: Orbitale; A: Point A (subspinale); ANS: Anterior Nasal Spine; Go: Gonion; Me: Menton; Gn: Gnathion.

The study sample was made up of 60 children, among whom 30 children with a confirmed diagnosis of intrauterine growth restriction (IUGR) and 30 healthy children were included. The groups were comparable in age (IUGR: 5.6 ± 1.0 years; Controls: 5.6 ± 1.1 years; p = 0.94) and sex distribution (52% female, 48% male; p = 1.00). Intra-examiner reliability for cephalometric measurements was excellent (ICC = 0.93; range 0.89-0.96), and inter-examiner agreement for dental scoring was high (κ = 0.86). Figures 1, 2 illustrate typical anteroposterior and frontal cephalometric radiographs with the landmarks identified for measurement. The analysis of cephalometric data showed that the groups significantly differed from each other (Table 1). Comparatively, the IUGR group showed a statistically significant reduction in the length of the cranial base (91.3 ± 4.9 mm) compared to the control group (99.6 ± 5.4 mm, p < 0.001) as well as in the mandibular length (65.2 ± 4.0 mm vs. 73.9 ± 4.5 mm, p < 0.001). At variance with the above, the lower facial height in the IUGR group was significantly higher than that of the controls (54.6 ± 3.1 mm vs 51.0 ± 3.2 mm, p < 0.001). These data hint at the occurrence of disproportionate vertical growth patterns in children with IUGR.

**Table 1:** Craniofacial morphometric parameters in IUGR versus control children. Statistical note: Values represent mean ± standard deviation. An independent samples t-test (two-tailed) was applied; the level of significance was set at α = 0.05. p < 0.001 indicates a highly significant difference. Effect size interpreted as per Cohen’s d (0.2 = small, 0.5 = medium, 0.8 = large). IUGR: Intrauterine growth restriction

Parameter	IUGR Mean ± SD	Control Mean ± SD	t value	p-value	Effect size (Cohen’s d)
Cranial base length (mm)	91.3 ± 4.9	99.6 ± 5.4	−6.25	< 0.001	1.61 (large)
Mandibular length (mm)	65.2 ± 4.0	73.9 ± 4.5	−7.97	< 0.001	2.06 (very large)
Lower facial height (mm)	54.6 ± 3.1	51.0 ± 3.2	4.42	< 0.001	1.14 (large)

**Figure 3:**
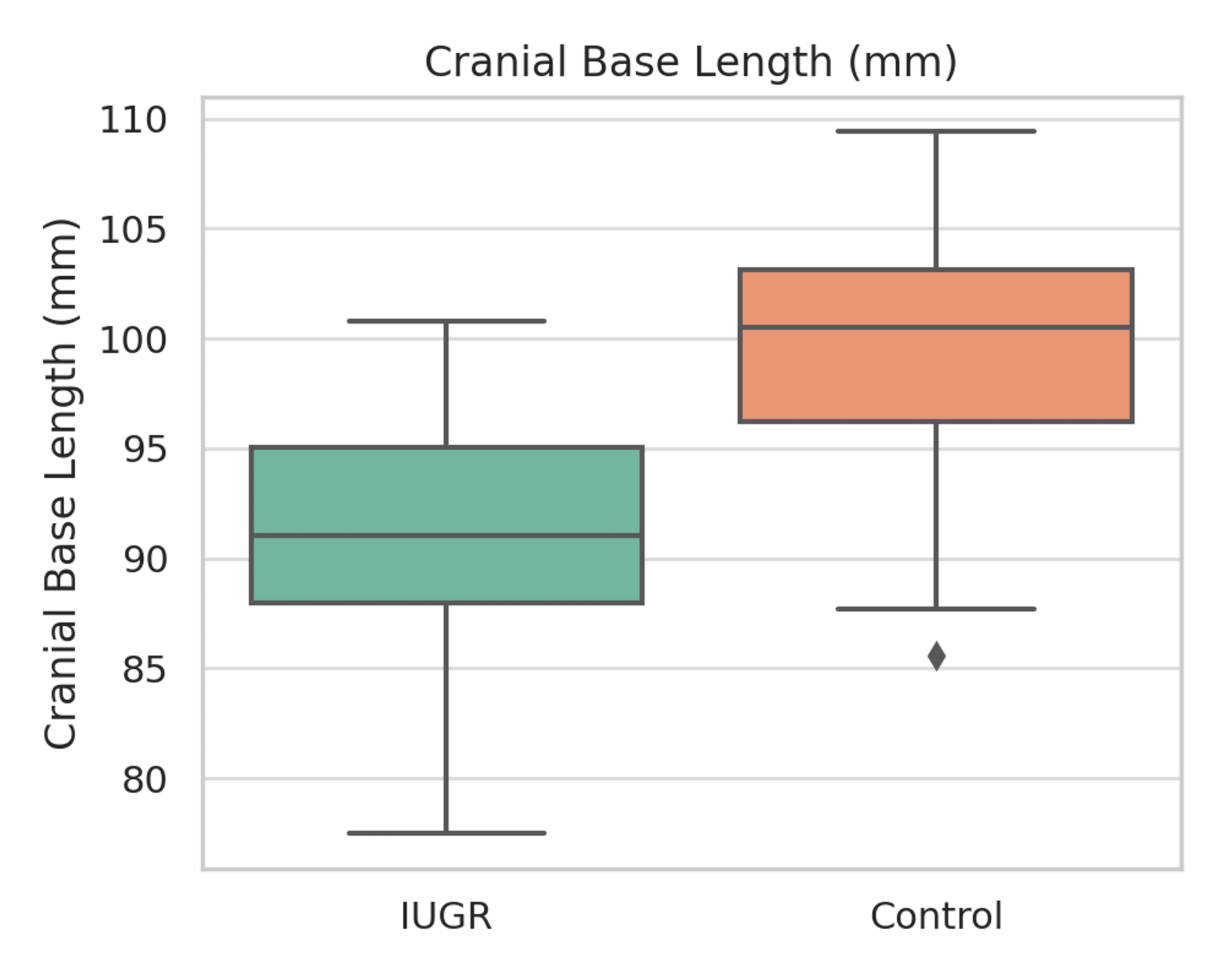
Boxplot of cranial base length (mm) in IUGR vs. control groups. Boxplot showing comparison of cranial base length (mm) between IUGR (n = 30) and control (n = 30) groups. The median cranial base length was significantly shorter in the IUGR group (t = −6.25, p < 0.001, Cohen’s d = 1.61). IUGR: Intrauterine Growth Restriction; mm: millimeter.

**Figure 4:**
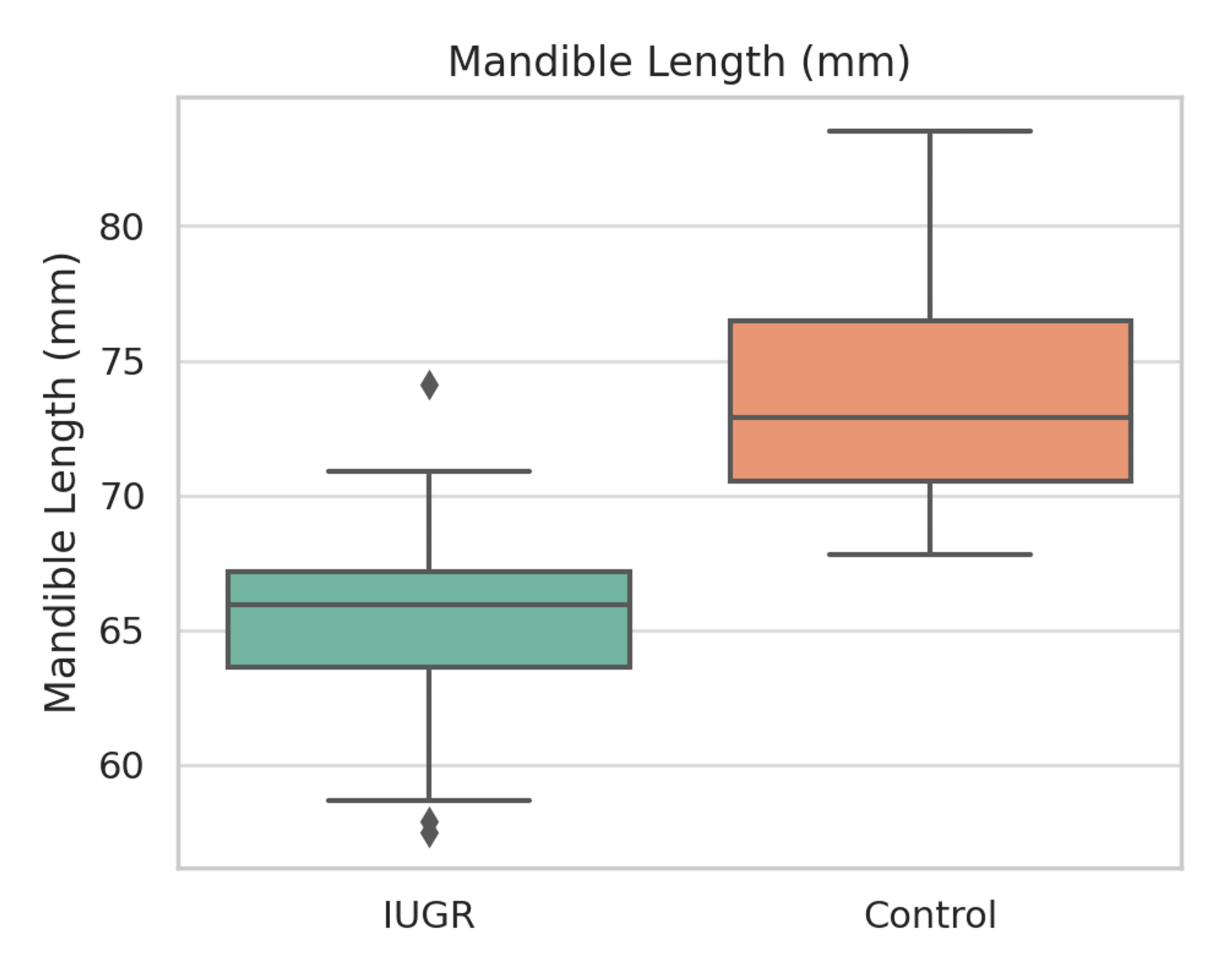
Boxplot of mandible length (mm) in IUGR vs. control groups. Boxplot showing comparison of mandibular length (mm) between IUGR (n = 30) and control (n = 30) groups. Mandibular length was significantly shorter in IUGR subjects (t = −7.97, p < 0.001, Cohen’s d = 2.06). IUGR: Intrauterine growth restriction; mm: millimeter.

**Figure 5:**
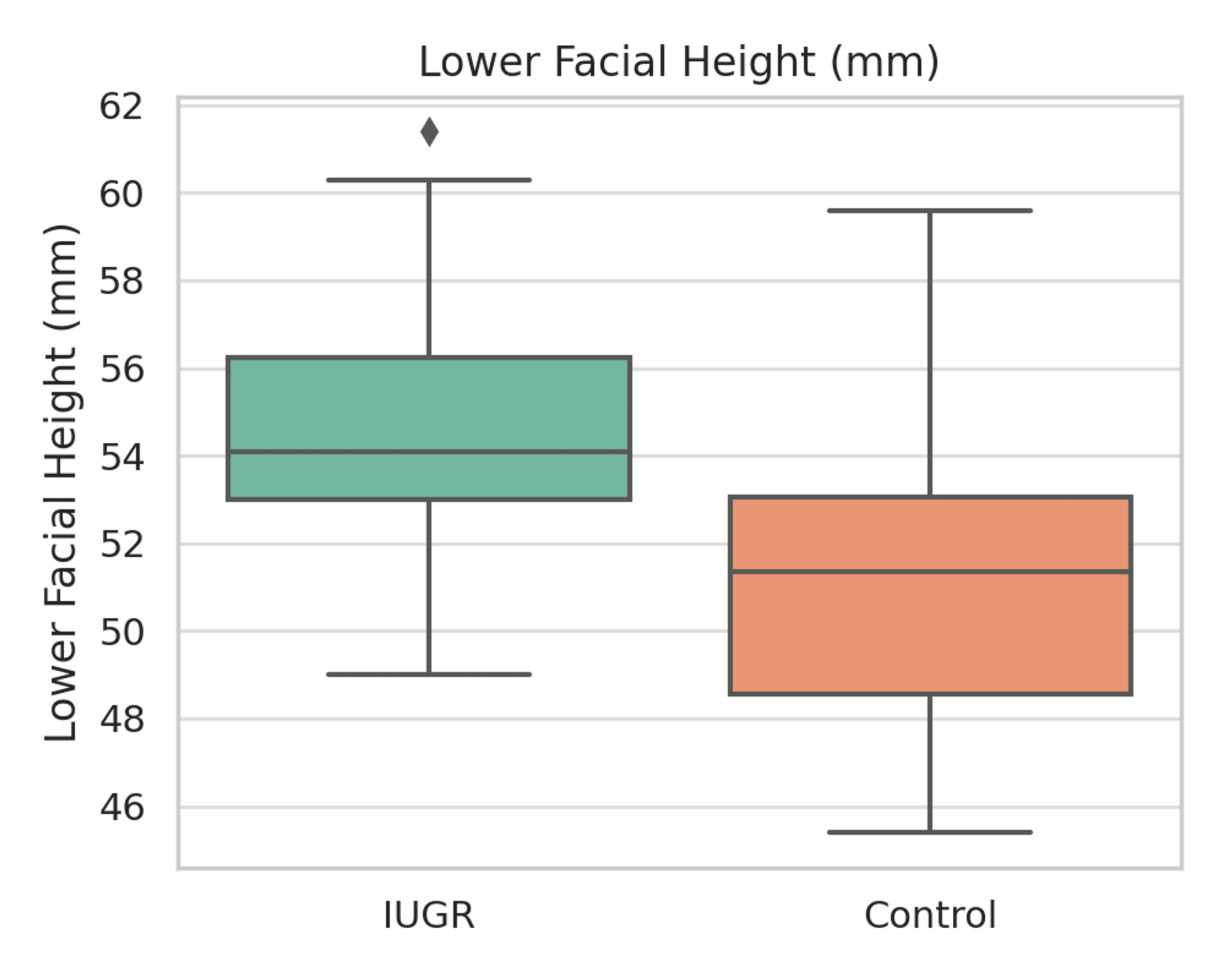
Boxplot of lower facial height (mm) in IUGR vs. control groups. Boxplot showing comparison of lower facial height (mm) between IUGR (n = 30) and control (n = 30) groups. Lower facial height was significantly greater among IUGR subjects (t = 4.42, p < 0.001, Cohen’s d = 1.14). IUGR: Intrauterine growth restriction; mm: millimeter.

Table 2 presents a summary of dental findings. Ten (33.3%) of the IUGR children were found to have enamel defects, whereas the incidence of such defects among the healthy children was only 3 (10.0%). Cramér’s V was calculated to be 0.21 (p = 0.06). The prevalence of caries (60.0% vs. 46.7%), hypodontia (20.0% vs. 20.0%), and microdontia (16.7% vs. 3.3%) was higher in the IUGR group, but the differences between the groups did not reach statistical significance (p > 0.05). Figures 3-5 illustrate boxplots showing significant differences in cranial base length, mandibular length, and lower facial height between the groups (p < 0.001).

**Table 2:** Prevalence of dental abnormalities among IUGR and control groups. IUGR: Intrauterine growth restriction; χ²: Chi-square test; p: Probability value. Statistical note: Values presented as n (%). Chi-square test applied (α = 0.05). p < 0.05 = significant. Cramér’s V is interpreted as 0.1 small, 0.3 medium, and 0.5 large effect.

Abnormality	IUGR (n = 30)	Control (n = 30)	χ²	p value	Cramér’s V
Caries	18 (60.0%)	14 (46.7%)	0.60	0.44	0.00
Hypodontia	6 (20.0%)	6 (20.0%)	0.00	1.00	0.00
Microdontia	5 (16.7%)	1 (3.3%)	1.67	0.20	0.10
Enamel defects	10 (33.3%)	3 (10.0%)	3.54	0.06	0.21

**Figure 6:**
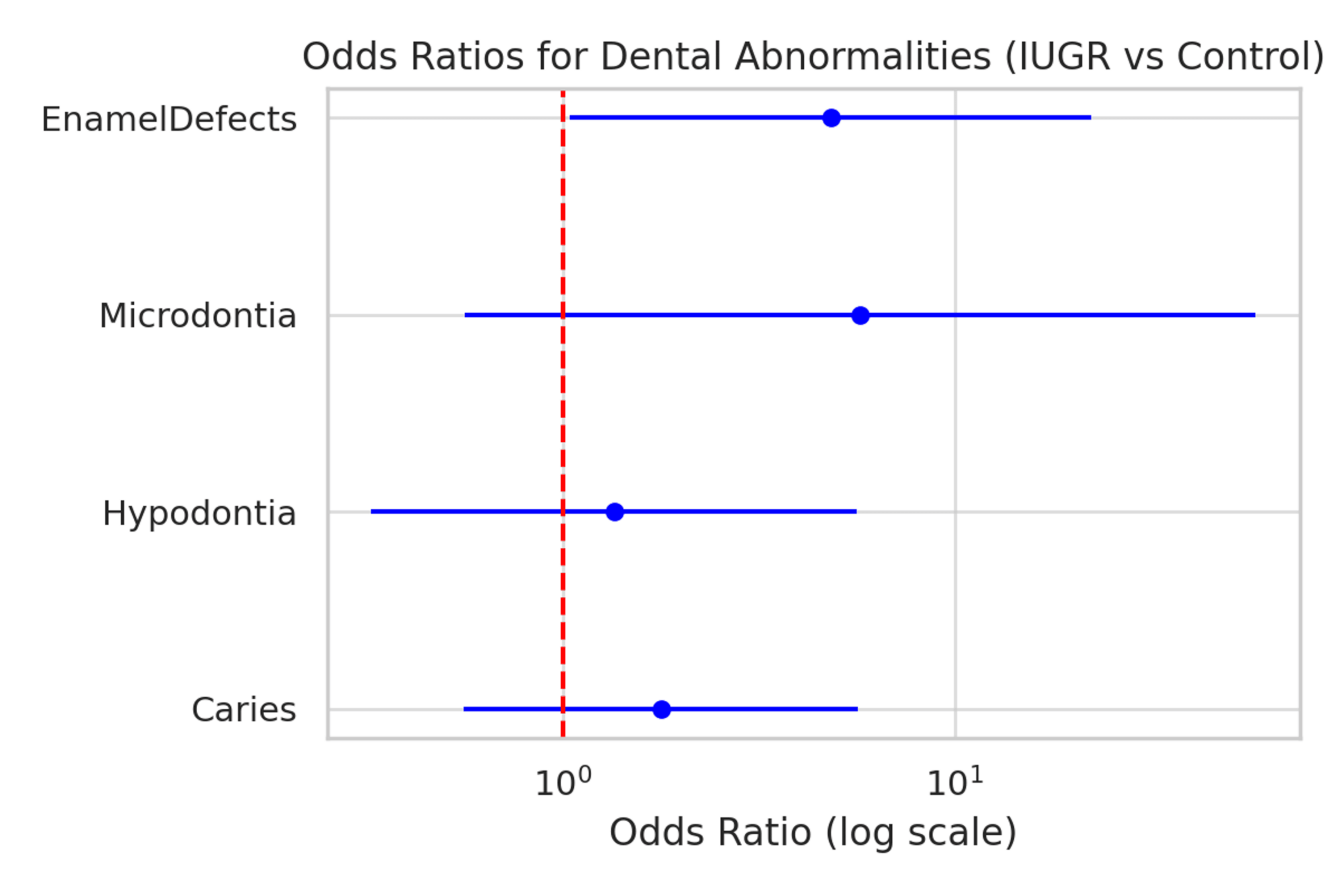
Forest plot of odds ratios for dental abnormalities in IUGR vs. control children. Forest plot illustrating odds ratios (95% CI) for dental abnormalities in IUGR (n = 30) versus control (n = 30) groups. The red dashed line marks OR = 1 (null value). Enamel defects (10 [33.3%] in IUGR vs. 3 [10.0%] in controls) showed a significant association (OR = 4.83, 95% CI = 1.04 to 22.36, p = 0.044). Other abnormalities were not significant. OR: Odds Ratio; CI: Confidence Interval; IUGR: Intrauterine Growth Restriction.

In order to ascertain the predictors of dental abnormalities, a multivariate logistic regression analysis (Table 3) was performed, along with controlling for age, sex, and socioeconomic status. The results show that after the adjustment, the presence of enamel defects was significantly related to IUGR (OR = 4.83, 95% CI 1.04-22.36, p = 0.044), which indicates that the probability of the occurrence of enamel hypoplasia in growth-restricted children is almost five times higher than in the normal group. Figure 6 shows a forest plot of adjusted odds ratios for dental abnormalities, indicating the strong association of IUGR with enamel defects.

**Table 3:** Binary logistic regression showing odds of dental abnormalities in IUGR subjects relative to controls. OR: Odds Ratio; CI: Confidence Interval; IUGR: Intrauterine Growth Restriction. Statistical note: Values are n (%) unless stated. Logistic regression applied; p < 0.05 = significant. An asterisk (*) denotes significance.

Outcome	IUGR (n = 30)	Control (n = 30)	OR (95% CI)	p-value	Interpretation
Caries	18 (60.0%)	14 (46.7%)	1.78 (0.56 – 5.65)	0.330	Not significant
Hypodontia	6 (20.0%)	6 (20.0%)	1.35 (0.32 – 5.60)	0.681	Not significant
Microdontia	5 (16.7%)	1 (3.3%)	5.75 (0.56 – 58.70)	0.140	Not significant
Enamel defects	10 (33.3%)	3 (10.0%)	4.83 (1.04 – 22.36)	0.044 *	Significant increase in IUGR group

Overall, these findings suggest that kids suffering from IUGR show significant changes in their craniofacial morphometrics, i.e., they have a shorter cranial base and mandibular dimensions, but their lower facial height is increased. Besides, they have a greater probability of enamel developmental defects.

## Discussion

The objective of the research was to compare craniofacial morphometry and the prevalence of dental abnormalities in children diagnosed with intrauterine growth restriction (IUGR) versus healthy, age-matched controls.

As a result, the IUGR group displayed two major changes: transformation of craniofacial skeletal dimensions and a plentifully increased chance of enamel developmental defects. These results highlight the long-lasting effect of limited prenatal development on the maxillofacial complex and are consistent with the references that describe the effects of somatic growth retardation in childhood [14-16].

Craniofacial skeletal alterations

The morphometric data revealed significantly lower cranial base length (91.3 ± 4.9 mm vs. 99.6 ± 5.4 mm; p < 0.001), shortened mandibular length (65.2 ± 4.0 mm vs. 73.9 ± 4.5 mm; p < 0.001), and increased lower facial height (54.6 ± 3.1 mm vs. 51.0 ± 3.2 mm; p < 0.001) in the IUGR children as compared to the controls. The combination of reduced cranial and mandibular dimensions, along with the vertically extended facial profile, characterizes the previously reported craniofacial features of children with generalized growth delay [14].

Davidopoulou and Chatzigianni found that children with growth retardation typically have shorter cranial base and mandibular lengths and increased lower anterior facial height, thus indicating that vertical skeletal divergence might be the most typical feature of limited somatic growth [14]. In the same vein, cephalometric studies of short children born small for gestational age (SGA) revealed that their craniofacial measurements were uniformly reduced except for lower facial height, thus suggesting that these disproportionate features have a prenatal origin and continue after birth [15]. Allareddy et al. documented similar evidence in growth-related syndromes, thus confirming that defective somatic growth is a primary factor leading to changes in craniofacial morphology [16]. SGA and IUGR together are a significant public health problem that causes the condition in approximately 2.5-3% of newborns worldwide, out of which up to 10% do not show catch-up growth. The consequence of this is persistent short stature, change in craniofacial proportions, and long-term developmental sequelae [17,18]. Thus, the current findings serve as an indicator that the development of the craniofacial skeleton is among the many systemic effects of IUGR.

Dental anomalies

In addition to skeletal changes, the present research determined enamel developmental defects as the most frequently occurring dental abnormality in the IUGR group of children (33.3% vs. 10.0%; p = 0.044, adjusted OR = 4.83). The identified relationship supports the work of Pinho et al., who found a significant correlation between IUGR and enamel hypoplasia [19]. Similarly, Garmash and Ryabokon revealed that dental anomalies' prevalence was higher in the case of children with symmetrical IUGR in the past, thus explaining that severe prenatal disturbances can lead to the interruption of hydration [7].

Although caries (60.0% vs. 46.7%), hypodontia (20.0% vs. 20.0%), and microdontia (16.7% vs. 3.3%) were more common in the IUGR cohort, these results did not reach statistical significance. This is a point opposite to the findings of Saraiva et al., who suggested that the prevalence of caries in IUGR children was lower, and they attributed it to the delayed eruption or early antibiotic exposure [18,19]. The lack of significance for hypodontia and microdontia may be due to the small number of samples and the cross-sectional nature of the study.

Implications for dental development and neurocognition

IUGR is a factor that changes the shape of the face and the skull and, at the same time, it affects the toot and neurodevlopment. Garg et al. revealed that the average age at which the first deciduous teeth appeared in IUGR infants (12.88 months) was significantly later than in the non-IUGR ones (6.9 months), thus providing more evidence for the association between prenatal growth restriction and delayed dental maturation [1]. Moreover, Hartkopf et al. found that the Mental Development Index (MDI) was significantly lower in the former IUGR infants at the age of two years compared with those born appropriate for gestational age, hence implying cognitive vulnerabilities occurring in conjunction with dental and skeletal delays [3].

These same mechanisms have been shown in ischemia and growth restriction during organ development experiments - in this case, hypoxia and growth restriction in the intrauterine environment disrupted chondrogenic signaling pathways, notably IGF-1 and BMP-mediated morphogenesis, resulting in changes in the development of craniofacial cartilage and bone [20,21].

The dental changes, in particular, enamel defects and microdontia, might be the delayed enamel mineralization of the developing enamel in the late gestation or infancy period. The placental insufficiency and hypoxia caused by IUGR can lead to dysfunction of ameloblasts and be worsened by the limited calcium-phosphate deposition process, ultimately resulting in hypoplasia and delayed eruption.

These data demonstrate that IUGR is linked to the changes in craniofacial development, which include the decrease in the length of the cranial base and mandibular dimensions, along with the increase in the lower facial height. Longitudinal follow-up is, therefore, necessary to determine whether such patterns remain in adolescence because, over time, compensatory catch-up growth may alter facial proportions.

Clinical implication

Early orthodontic monitoring of children with IUGR can result in the timely detection of skeletal discrepancies and the prevention of malocclusion progression.

Caution in interpretation

The association of IUGR with enamel defects (OR = 4.83; 95% CI 1.04-22.36) should be viewed cautiously, as the confidence interval is wide, which indicates that the interval is more variable and there are fewer events.

Limitations

A slight difference in the growth phase may still affect the outcomes, despite that the individuals were closely matched by age and sex. The study population, which was consecutively sampled from a single center, may not be representative of the general population, and the sample size is only adequate to provide the power for the secondary dental outcomes. More extensive multicenter, longitudinal studies are needed to confirm the direction of the relationship and developmental persistence.

## Conclusions

IUGR is linked to unique changes in the craniofacial morphometric features, the length of the cranial base and the mandible being shorter, and the lower facial height being increased, together with a greater frequency of enamel defects. The links here signal how facial development after birth is affected by prenatal growth limitation, and thus, they convey the necessity of orthodontic check-ups and pediatric growth monitoring next in line. Due to the cross-sectional nature of the study and the small sample size, the findings should be considered as associations only and not as cause-and-effect relationships.

## Appendices

**Table 4:** Demographic matching of IUGR and control participants An anonymized dataset showing age- and sex-matched pairing between IUGR and control participants. No statistically significant difference was found in age or sex distribution between groups (p > 0.05).*Socio-economic status was classified by Kuppuswamy’s scale based on parental education and occupation.

Participant ID	Group (IUGR/Control)	Sex (M/F)	Age (years)	Socio-economic status*
C01	Control	F	3.4	Middle
I01	IUGR	F	3.5	Middle
C02	Control	M	3.8	Middle
I02	IUGR	M	3.7	Middle
C03	Control	F	4.0	Lower-Middle
I03	IUGR	F	3.9	Lower-Middle
C04	Control	M	4.2	Middle
I04	IUGR	M	4.1	Middle
C05	Control	F	4.5	Upper-Middle
I05	IUGR	F	4.6	Upper-Middle
C06	Control	M	4.7	Middle
I06	IUGR	M	4.8	Middle
C07	Control	F	5.0	Middle
I07	IUGR	F	5.0	Middle
C08	Control	M	5.2	Lower-Middle
I08	IUGR	M	5.3	Lower-Middle
C09	Control	F	5.4	Middle
I09	IUGR	F	5.5	Middle
C10	Control	M	5.6	Middle
I10	IUGR	M	5.6	Middle
C11	Control	F	5.8	Upper-Middle
I11	IUGR	F	5.9	Upper-Middle
C12	Control	M	6.0	Middle
I12	IUGR	M	6.1	Middle
C13	Control	F	6.2	Middle
I13	IUGR	F	6.2	Middle
C14	Control	M	6.4	Lower-Middle
I14	IUGR	M	6.3	Lower-Middle
C15	Control	F	6.6	Middle
I15	IUGR	F	6.7	Middle
C16	Control	M	6.8	Middle
I16	IUGR	M	6.9	Middle
C17	Control	F	7.0	Upper-Middle
I17	IUGR	F	6.9	Upper-Middle
C18	Control	M	7.1	Middle
I18	IUGR	M	7.0	Middle
C19	Control	F	7.2	Lower-Middle
I19	IUGR	F	7.3	Lower-Middle
C20	Control	M	7.4	Middle
I20	IUGR	M	7.4	Middle
Mean ± SD			5.6 ± 1.1 yrs (Control) / 5.6 ± 1.0 yrs (IUGR)	
Sex distribution		M = 14 (46.7%), F = 16 (53.3%) in each group		
